# A qualitative pilot study of food insecurity among Maasai women in Tanzania

**Published:** 2012-07-23

**Authors:** Carol Fenton, Jennifer Hatfield, Lynn McIntyre

**Affiliations:** 1 Undergraduate Medical Education, Faculty of Medicine, University of Calgary, 3330 Hospital Drive, Calgary, Alberta, Canada; 2 Department of Community Health Sciences, Faculty of Medicine, University of Calgary, 3E14 TRW Building, 3280 Hospital Drive NW, Calgary, Alberta, Canada

**Keywords:** Food security, food supply, human rights, qualitative research, Eastern Africa

## Abstract

**Background:**

Food insecurity is an ongoing threat in rural sub-Saharan Africa and is complicated by cultural practices, the rise of chronic conditions such as HIV and land use availability. In order to develop a successful food security intervention program, it is important to be informed of the realities and needs of the target population. The purpose of this study was to pilot a qualitative method to understand food insecurity based on the lived experience of women of the Maasai population in the Ngorongoro Conservation Area of Tanzania.

**Methods:**

Short semi-structured qualitative interviews with 4 Maasai women.

**Results:**

Food insecurity was present in the Maasai community: the participants revealed that they did not always have access to safe and nutritious food that met the needs of themselves and their families. Themes that emerged from the data fell into three categories: Current practices (food sources, planning for enough, food preparation, and food preservation), food Insecurity (lack of food, emotions, coping strategies, and possible solutions), and division (co-wives, food distribution, and community relationships).

**Conclusion:**

This pilot study suggested the presence of food insecurity in the Maasai community. Larger sample studies are needed to clarify the extent and severity of food insecurity among this population. Having a detailed understanding of the various aspects of the food insecurity lived experience could inform a targeted intervention program.

## Background

In 2000, the United Nations General Assembly declared a goal to reduce by half the proportion of people who suffer from hunger by 2015 [1, 2]. Some progress has been made in achieving the hunger reduction goal, however, the countries that have seen the least progress towards the hunger-reduction goals are in South Asia and sub-Saharan Africa [2].

The 1996 World Food Summit defined food security as “when all people, at all times, have physical, social and economic access to sufficient, safe and nutritious food that meets their dietary needs and food preferences for an active and healthy life” [3]. Thus food security encompasses inadequate diets, unsustainable food production, inappropriate foods, and socially unacceptable ways in which people secure food, and encompasses many more than the 923 million people counted by the FAO.

A person's or household's food security status rests upon three pillars: 1) food availability or adequate food production; 2) economic access to available food; and 3) utilization or nutritional security, which includes short- and long-term aspects [4–6]. In order to be considered food secure, people must have access to sufficient and high-quality food at all times In this context, food insecurity is a lack of food security and encompasses more than chronic food deprivation or hunger.

The Maasai people traditionally roamed with their cattle throughout southern Kenya and northern Tanzania [7] in a transhumanist pastoral lifestyle [7–9]. Traditionally the Maasai herded cattle over large distances in order to make use of sparse resources such as pastures and water. Since colonization and land privatization, the Maasai can no longer roam much of their traditional pasture land. The Ngorongo Conservation Area (NCA) is a national park that has remained natural landscape, so a large number of Maasai in Tanzania have moved to the NCA to use the natural space to practice traditional herding. The NCA is currently balancing conservation, tourism, and agropastoralism. These conflicting interests are often a source of controversy [10, 11]. For example, there is a great concern in regards to allowing people to cultivate in the NCA, for fears that the wild animals and tourists will be disturbed. However, cultivation is important for the Maasai to survive: Grain, both subsistence-grown and purchased, now plays a major role in the Maasai diet, as milk yields decrease with the onset of the dry season [10, 12, 13]. For example, when the outright ban on cultivation in the NCA was lifted in 1995, severe child malnutrition among the local Maasai declined from 19% to 3% [10, 13]. Currently, the Maasai are permitted to practice small-scale subsistence agriculture, provided ploughs are not used [10].

The purpose of this pilot study was to assess the feasibility of using semi-structured informant interviews and ethnographic observation to gain an understanding of the lived experience of food insecurity among the Maasai in the NCA of Tanzania.

The biggest debate in food security measurement is the general approach: whether to use quantitative or qualitative research methods. While quantitative methods allow the researcher a broad picture of the frequency and severity of food security, qualitative methods give a more in-depth understanding of what it means to be food secure or food insecure [14].

Many researchers recognize the value of both approaches, and advocate for the application of both or mixed methods [14]. The most common methods for measuring food security include consumption measures, which look at calorie and nutrient intakes; survey-based modules such as the US Household Food Security Survey Module (HFSSM); and experience-based measures. Consumption measures are not only expensive and time-consuming, they capture only the physiological sufficiency of food intake [15]. The HFSSM, being a quantitative survey measure, is useful for assessing prevalence levels of food security to target population-level intervention programs, as well as monitoring prevalence over time for the impact of interventions and program evaluations [14]. Some researchers claim that rich detail from qualitative analysis of particular human behaviours is lost when reduced to statistical measures [14].

Experience-based food security measures are used to advance understanding of the specific behaviours and coping mechanisms associated with food insecurity. This qualitative approach incorporates more of the elements of the complex theoretical construct of food security beyond consumption measures [15]. The experience-based, qualitative approach has the intention of understanding the perceptions and behaviours of the people who are experiencing food security or food insecurity [16]. Finally, the results of the experience-based measure could be used to develop a more quantitative approach (similar to the US HFSSM) that is specific to this community's needs [16].

## Methods

A semi-structured interview guide was adopted from a concurrent study conducted in Bangladesh [17] and translated into Maa, the native Maasai language. Because women have central roles in household-level food provisioning, they were sought as 'potential participants for the study through word of mouth among women staying at or near the Endulen Hospital, in the NCA. The women who volunteered for the study self-selected, and were not hospital patients. The sample size for this small pilot project was 4 women. A small sample size is advantageous when applying a qualitative, ethnographic method: a greater amount of time can be spent with each participant to achieve depth and richness in the data collected [18]. The method sought to obtain detailed, descriptive accounts of women's everyday experiences and practices of providing for and managing their family's food resources. It also sought to provide women with an opportunity to respond and comment on current interventions, as well as provide ideas and feedback for the development of future interventions to help address this problem. The voices of the four women are not meant to be representative nor did we learn everything about Maasai women's experience of food insecurity, a term known as saturation in qualitative methods; rather, these interviews provide insights and experiences that would not be elucidated by a survey. Furthermore, the purpose of a pilot study is to evaluate the ability of the research tool to access the data sought by the research question. Once the utility of the tool has been evaluated, a larger number of interviews may be undertaken to increase the depth of understanding on the topic.

The interview tool was developed to be specific to local concerns, and the interview questions were developed with information from key informants working in the health system and living locally. The data were analyzed in conjunction with interviewer reflections and observations recorded at the time of the interview, which were also entered into the dataset. The interviews were digitally recorded, transcribed, and translated into English. The qualitative interview themes were refined through focus groups with members of the community. Textual segments of the pilot interview transcripts were coded using the qualitative analysis program, NVivo. These codes were used to generate a list of common themes. Once the electronic analysis was complete, the transcripts were reviewed in their entirety for immersion.

## Results

The sample size for this small pilot project was four women, who all lived within the NCA. The women were all married, and varied in age and number of children (Table 1). The following themes emerged from the data (which are organized into three general categories: Current Practices (Food sources, Planning for enough, Food preparation, Food preservation), Food Insecurity (Lack of food, Emotions, Coping strategies, Possible solutions), and Division (Co-wives, Food distribution, Community relationships). At least two out of the four women mentioned each theme which are described more fully below.

**Table 1:** Socio demographic characteristics of respondents

Respondents	Approximate Age (years)	Number of dependent children
1	35	3
2	60	8
3	26	0
4	28	4

### Current Practices

This category includes strategies that a Maasai woman uses to plan for, source, and store food for herself and her family's use. The themes included in this category are Food sources, where the Maasai women talked about the different sources they accessed to procure food; Planning for enough, where the women discussed how they plan to achieve access to food in the future; Food preparation, where the women described their food preparation process; and Food preservation, where the women talked about storing food for future use.

### Food Sources

The Maasai women reported that their primary sources of food were milk from their cattle and maize from their gardens. While they also reported accessing potatoes, rice, meat, and beans, in fact, enough food was understood to mean enough milk as illustrated by the following quotes from participants:

“It is just to have a lot of cattle in order to get milk from them.” “Sometimes during rainy seasons we depend on milk only and dry season we can buy potatoes and flour.”

Subsistence agriculture also played a role:

“Always people use to dig farms in order to get flours. I did myself the farm…If there is no farm, then I sell a cow.”

### Planning for enough

The women described estimating corn harvests:

“You dig a farm and harvest then you can approximate that maybe this maize are enough until next year.”

The women also highlighted the importance of the herd to Maasai food provisioning:

“I have to keep cattle so I may not miss food, because a Maasai food is cow. If you don't have cow, you won't get food.”

### Food Preparation

To gain insight into the Maasai food experience, and to gain an understanding of Maasai dietary practices, we asked the women to describe the meal preparation process. The women described the frequency of meals:

“Three times. You start tea, then porridge, then ugali (cornmeal) at the afternoon for children…

Because children need to eat at morning, afternoon, and evening. And this depends on the food you have. If you have less then you cook two times.”

The women also described what types of food they were typically consuming: “If cows are many I have to milk them then store the milk in the culabush (gourd) to be saved for my husband or I can keep for somebody else we are related and if we do have a plenty of milk then we will leave flour. Children will depend on milk only and all people and if there is no milk, then I cook porridge by putting water into the pot and put on the fire to boil and mix with flour, milk, fat if you have, salt. Then you take off from the fire and ready for drink.”

The type of meal the women described most frequently was porridge:

“You start by putting water on the fire, boiling maize with flour. If it is your planning and you add milk, boiling together and give some minutes boiling then after you take off the fire then it is ready for drink.”

One woman stated that that porridge was a typical Maasai meal, but members of the community were beginning to cook other foods as well:

“We Maasai cook porridge but nowadays there is change because people cook tea, ugali (cornmeal), and beans, other people cook three times a day and other two times a day and so on.”

### Food preservation

Three out of four women reported various ways of storing maize for the future, either in sacks, at the store in town, or in an orliitanet:

“We do have small store call orliitanet (a place built by sticks which you can put maize without using bags).”

### Food Insecurity

The category “Food Insecurity” includes the themes that indicate the presence of food insecurity in the Maasai community. The themes in this category include Lack of food, where women describe instances where they experienced food shortages; Emotions, where women describe how they feel about their food situation; and Coping strategies, where the women describe strategies that they employ in order to procure food for themselves and their families.

### Lack of food

Food shortages were real and often seasonal:

“There is a year which have no rain and if there is not enough rain then the farms cannot grow well, and also for the cows, bull never met with cows, which means they don't give birth and if you don't have food at all then you can ask from somebody else who have. If you miss food from the farms and you have cows, then it is help – than having nothing.”

“There are actually good years and bad years which there is no food and cows are not producing milk, so it depends on season.”

While the women reported sharing relationships that help them mitigate food shortages, inequalities in herd size highlighted social inequalities in the community:

Interviewer: “Is there any other time which is harder to feed your family?”

“Yes, that is why I told you from in the beginning that people are not equal by cattle, for that reason there is other small village which have not enough cattle.”

### Emotions

The women expressed worry and concern regarding their ability to provide food for themselves and their children:

“We all worried, everybody who have children is worried. All who bearing children.”

“It is bad because if you don't have food for your children you feel bad, a big worry is lack of food for children. It is confusing if there is no food.”

### Coping Strategies

The women described various strategies they employed when they did not have enough food. These strategies included providing children with tea:

“Yes, but if you miss food, then you can cook tea for children”; Doing without food:”Nothing to do out of stay only;”

seeking help from relatives or neighbours:

“Maybe my mother can find me food and if she miss then she can come stay with us.” Other strategies included selling cows from their herd to buy food, and sending the Morani, or young adult males, to earn money to buy food:

“… if you have young Morani, they can go to work and buy us food. But if you don't have Morani, it is a big problem.”

“And if there is no rain, then the young people who are making business of buying and selling things can bring us food.”

### Possible solutions

We asked the women to think of solutions to any food security issues they encountered. We received a wide range of responses to this question, from no answer, to seeking handouts, to working with the government to find solutions, to entrepreneurship:

Interviewer: “What are you thinking, how do people help you to get food which can be enough for the family?

“I like to get help and I like to be together with government for whatever they give us. So, I am not thinking it is bad for someone who can help me.”

“It just people to be given maize by government or NGOs” (Non-Governmental Organizations)

“What I will be thinking especially for children is that, how can I get a business of selling knives in order to get money for flour and make the business of selling firewood and there are so many Maasai thinking.”

**Division** The category “Division” includes the themes that encompassed the nature of food distribution and sharing in the Maasai community. These themes include Co-wives, in which the respondents talk about the division of food and labour among Maasai women married to the same man; Food distribution, which yields insight into the division of food between members of a Maasai family, and Community relationships, which describe the nature of division and sharing of food within the Maasai community.

### Co-wives

The presence of this theme highlights the unique cultural, labour and resource structures within the Maasai community. Two of the respondents discussed division of labour and food between co-wives. As one woman explained:

“If I have co-wives, our husband can give us one such of maize and we share by dividing to each one equally”

### Food distribution

Within a Maasai household (olmarei), the food is divided evenly among wives. Each wife must then provide for her own children. This quote highlights the vulnerability of Maasai women, as they are last to be served:

“After cooking, I have to wash my cups and put porridge for each child but I have to give first my husband and then children after. And if I miss porridge than I can get little remain at the bottom of the pot”

### Community relationships

Three of the Maasai women interviewed mentioned the importance of relationships in the community. The women explained that food was shared between neighbours in times of need:

“We used to ask, if there is someone went to grind maize at the machine, then you visit and ask a little amount of flour to cook for your children, then she can give you a small cup.”

## Discussion

The findings of this study suggest that food insecurity is present in the Maasai community as defined by the World Health Organization because these participants shared that they did not always “have physical, social and economic access to sufficient, safe and nutritious food that met their dietary needs and food preferences for an active and healthy life” [3]. These Maasai women were not always able to ask their husbands to sell a cow to buy food; there was a lack of social access to food when the neighbours were unable to share their food; and there was a lack of sufficient food, as the women reported experiencing hunger during the dry season. Furthermore, the women reported several coping strategies they developed in order to mitigate the impact of food insecurity. The mere presence of coping strategies may indicate the presence of food insecurity [15].

The results of this study highlight the uniqueness of the Maasai community and their social structures. Maasai communities differ from other populations that may be experiencing food insecurity in two key ways: the nature of Maasai polygamous marriage structures as well as the tight-knit community relationships. Although the Maasai co-wives cooperate in the division of household labour and share food supplies, food is also equally divided among them, which may limit individual amounts. Furthermore, the practice of sharing food within the community is important as three out of the four women interviewed reported food sharing as their main coping strategy when food supplies were limited.

For Maasai women in the NCA, food supplies are highly variable and subject to seasonality. During the dry season, lower milk production from cows and low stores of maize led to food insecurity. Food insecurity is exacerbated by inequalities such as variations in herd size. Herd size is very important as cattle can act as a buffer against food insecurity. For example, if the Maasai have many cattle, they are able to subsist primarily on milk, and thus are able to spare more grain for later. Furthermore, if they have many cattle, they can choose to milk less, leaving more for the calves and increasing the calves’ chance of survival, which may improve herd health in future [19]. Also, if the cows stop producing milk and the family runs out of maize, it is feasible for those who have large herds to sell a cow and use the profit to purchase food.

The coping strategies reported by the women interviewed suggest the need for intervention, as they are not sustainable for long-term food security. For example, one coping strategy was to seek food from neighbours. However, having to rely on neighbours for food is problematic because of the need for reciprocity, and the likelihood of being able to provide for other families over the long-term is questionable, particularly in times of limited food supplies. Furthermore, if an individual goes to visit another boma (household) for food, the chances of sexual encounters are then increased, putting individuals at risk of contracting the HIV virus [20]. Another coping strategy identified in the interviews that enables women to procure food in times of food insecurity was to sell a cow from their herd. This strategy is problematic because the sale of a cow represents a decrease in the overall herd size, and thus a decrease in the family's buffer against future food insecurity. Finally, one of the women reported sending Morani (young men from the community) to work in urban areas for money to buy food. This coping strategy could be problematic, as having to work to provide for their family not only increases the risk of contracting HIV but decreases the likelihood that these adolescent boys are able to pursue an education [21].

### Implications

**Need for research:** The results of this pilot study describe a situation of food insecurity among the Maasai people that illustrates an importance for further research in this area.

**Possible policy implications:** In order to deliver aid effectively, it is important for organizations to understand households’ objectives, attitudes, and options available to them to obtain food [17, 22]. When considering the design of a development or food program for the Maasai, their unique culture must be a key element of the design, in order for the programit to be applicable, feasible and sustainable [9, 17, 22].

The Maasai recognize organizations’ failure to engage them in development programs [7]. In the past, the Tanzanian government and various NGOs explored various policies for sedentarization of the Maasai. The unfolding of ujamaa (President Nyerere's national development project) and other development policies among the Maasai did not include consultation with the Maasai, and included little consideration of the existence and purpose of their specific cultural practices [13]. As a result of the perceived insensitivity of the government to their concerns, the Maasai expressed little faith in the government [7].

While the history of development efforts highlight the importance of engaging the Maasai community in development efforts, it is also important to take into consideration other stakeholders, particularly the Ngorongoro Conservation Area Authority. The results from this pilot study suggest that increasing herd size would help buffer Maasai families from food insecurity situations. However, this option represents only the needs of the Maasai. Increasing herd size may not be a feasible solution to Maasai food insecurity, because not only are there ecological limits on the number of cattle that can be sustained in the NCA, the NCA Association would likely be opposed to such a measure, as the cattle would represent a competition for resources for the natural wildlife. Considering tourism is one of the strongest industries in Tanzania, and a large proportion of government funding is from tourism, the NCA Association has a strong influence on policy and regulation within the NCA. Therefore, policymakers aiming to improve conditions for the Maasai must strive to work in cooperation with all stakeholders. According to Thornton et al., Maasai households could mitigate external food security stresses by increasing the size of their cultivated plots [11]. Increases in cultivation not only have implications for the NCAA, but also have implications for Maasai mobility. Private land ownership may be necessary in order to increase agriculture. Increased cultivation could represent a further decrease in traditional Maasai practices, which may be met with opposition in the Maasai community [7].

Maasai women participate less in the cash economy, which has been primarily attributed to lower levels of educational attainment [7]. Maasai women have been known to participate in small-scale activities such as selling surplus milk, making and selling of beadwork and minor shop-keeping. However, these activities tend to vary seasonally and reflect specific opportunities [7]. Furthermore, it has been recognized that the majority of Maasai are marginalized from the income-generating opportunities provided by increased tourism, an issue that affects both Maasai men and women [7]. The findings from this study suggest that Maasai women might be interested in increasing opportunities to diversify income. Therefore, introducing programs that would engage women in such activities could be a possible avenue to explore.

## Conclusion

In this pilot study, the Maasai women reported experiencing seasonal food shortages, worrying about providing for their families, and coping strategies to mitigate the impact of food shortages. These findings suggest the presence of food insecurity in the Maasai community, demonstrating the value of this qualitative approach. Food insecurity is a problem that needs to be defined and addressed because it monopolizes women's time and focus, decreasing available time for health promotion activities and may increase the risk of contracting HIV. To design feasible and successful intervention programs, more extensive research needs to be done in order to understand the nature of food insecurity in the Maasai community

## References

[1] UN

[2] UN The Millennium Development Goals Report 2008.

[3] WHO Food Security. http://www.who.int/trade/glossary/story028/en/.

[4] Quisumbing AR, Brown LR, Feldstein HS, Haddad L, Pena C Women: The Key to Food Security.

[5] USAID Definition of Food Security19Washington, D.C. http://www.usaid.gov/policy/ads/200/pd19.pdf.

[6] Swindale A, Bilinsky P (2006). Development of a Universally Applicable Household Food Insecurity Measurement Tool: Process, Current Status, and Outstanding Issues. Journal of Nutrition..

[7] Coast E (2002). Maasai Socioeconomic Conditions: A Cross-Border Comparison. Human Ecology: An Interdisciplinary Journal..

[8] Boone RB, Burnsilver SB, Thornton PK, Worden JS, Galvin KA (2005). Quantifying Declines in Livestock Due to Land Subdivision. Rangeland Ecology & Management..

[9] Forstater M (2002). Bones for Sale: ‘development’, environment and food security in East Africa. Review of Political Economy..

[10] Coast E (2002). Maasai Socioeconomic Conditions: A Cross-Border Comparison. Human Ecology: An Interdisciplinary Journal..

[11] Thornton PK, Boone RB, Galvin KA, Burnsilver SB, Waithaka MM, Kuyiah J (2007). Coping Strategies in Livestock-dependent Households in East and Southern Africa: A Synthesis of Four Case Studies. Human Ecology: An Interdisciplinary Journal..

[12] Nestel PS (1989). Food intake and growth in the Maasai. Ecology of Food and Nutrition..

[13] Fratkin E, Mearns R (2003). Sustainability and Pastoral Livelihoods: Lessons from East African Maasai and Mongolia. Human Organization..

[14] Coates J, Wilde PE, Webb P, Rogers BL, Houser RF (2006). Comparison of a qualitative and a quantitative approach to developing a household food insecurity scale for Bangladesh. J Nutr..

[15] Maxwell D, Ahiadeke C, Levin C, Armar-Klemesu M, Zakariah S, Lamptey GM (1999). Alternative food-security indicators: revisting the frequency and severity of ‘coping strategies’. Food policy..

[16] Habicht JP, Pelto G, Frongillo EA, Rose D Conceptualization and instrumentation of food insecuri, National Academy of Sciences Workshop.

[17] McIntyre L, Rondeau K, Kirkpatrick S, Hatfield J, Islam KS, Huda SN (2011). Food provisioning experiences of ultra poor female heads of household living in Bangladesh. Soc Sci Med..

[18] Denzin NK (2003). Lincoln YS: Collecting and Interpreting Qualitative Materials.

[19] Homewood K (1992). Development and the Ecology of Maasai Pastoralist Food and Nutrition. Ecology of Food and Nutrition..

[20] Coast E (2007). Wasting semen: context and condom use among the Maasai. Cult Health Sex..

[21] Morton J (2006). Conceptualising The Links Between Hiv/aids And Pastoralist Livelihoods. The European Journal of Development Research..

[22] Thornton P, Boone R, Galvin K, BurnSilver S, Waithaka M, Kuyiah J, Karanja S, González-Estrada E, Herrero M (2007). Coping Strategies in Livestock-dependent Households in East and Southern Africa: A Synthesis of Four Case Studies. Human Ecology..

